# International developments in revenues and incomes of general practitioners from 2000 to 2010

**DOI:** 10.1186/1472-6963-13-436

**Published:** 2013-10-24

**Authors:** Madelon Kroneman, Pascal Meeus, Dionne Sofia Kringos, Wim Groot, Jouke van der Zee

**Affiliations:** 1 NIVEL Netherlands Institute of Health Services Research, P.O Box 1568, 3500, BN Utrecht, The Netherlands; 2 RIZIV, 1150 Brussels, Belgium; 3 Department of Social Medicine, Academic Medical Centre, University of Amsterdam, Amsterdam, The Netherlands; 4 Department of Health Services Research, Faculty of Health Medicine and Life Sciences, Maastricht University, Maastricht, The Netherlands; 5 Department of International Health, Faculty of Health, Medicine and Life Sciences, Maastricht, The Netherlands

**Keywords:** General practice, Income, International comparative research

## Abstract

**Background:**

The remuneration system of General Practitioners (GPs) has changed in several countries in the past decade. The aim of our study was: to establish the effect of these changes on the revenues and income of GPs in the first decade of the 21^st^ century.

**Methods:**

Annual GP revenue and practice costs were collected from national institutes in the eight countries included in our study (Belgium, Denmark, Finland, France, Germany, The Netherlands, Sweden, The United Kingdom (UK)) from 2000–2010. The data were corrected for inflation and purchasing power. Data on the remuneration systems and changes herein were collected from the European Observatory Health Systems Reviews and country experts.

**Results:**

Comprehensive changes in the remuneration system of GPs were associated with considerable changes in GP income. Incremental changes mainly coincided with a gradual increase in income after correction for inflation. Average GP income was higher in countries with a strong primary care structure.

**Conclusions:**

The gap between the countries where GPs have a lower income (Belgium, Sweden, France and Finland) and the countries where GPs have a higher income (Netherlands, Germany and the UK) continues to exist over time and appeared to be related to dimensions of primary care, such as governance and access. New payment forms, such as integrated care payment systems, and new health care professionals that are working for GPs, increasingly blur the line between practice costs and income, making it more and more important to clearly define expenditures on GPs, to remain sight on the actual income of GPs.

## Background

The remuneration system of GPs provides incentives, intended and unintended, for GP behaviour. For instance, fee-for-service payments are expected to provide incentives for GPs to treat patients in their own practice as long as possible instead of referring them to other health care providers. With salary and capitation systems there will be a risk of unnecessary referrals to other (more costly) health care providers [1-5]. However, in practice, GPs also face non-financial incentives, ethical considerations and professional standards [6]. The incentives of payment systems probably get considerable attention, because they can be more easily changed by policy makers than other incentives [7]. During the past decade, new payment systems have been introduced, such as pay-for-performance and integrated care payments. Currently it is not known to what extent these changes have influenced the income of GPs. In a previous paper [8] on the remuneration and income of General Practitioners (GPs) covering the period from 1990 to 2005 in eight European countries (Belgium, Denmark, Finland, France, Germany, the Netherlands, Sweden and the United Kingdom), we showed that: 1) in some countries (Germany, Netherlands, UK) GPs earn more than in others (Belgium, France, Sweden), 2) these differences remained rather stable over time, and 3) the incomes of British GPs increased disproportionally after the 2004 remuneration reforms [8,9]. As we were aware that the UK was not the only country where remuneration reforms took place, we decided to update our study and to analyse the period 2000–2010 in more detail (using annual intervals instead of the 5-year intervals in our previous study) and to focus on the effects of remuneration reforms on the revenues and incomes of GPs. Our previous study [8] showed that gate-keeping GPs had a systematically higher income compared to GPs in countries where patients had direct access to secondary care. Data on GP’s tasks were not available at that time. Recently, a comprehensive European study into the strength of primary care has been published, and we have used these data to see whether a strong primary care sector is related to higher payments of GPs [10]. Differences in income levels of GPs in different countries may be related to the importance and the role of primary care in the health care system (we will refer to this as the strength of primary care). We expect that in health systems with a strong primary care, where general practice has a more comprehensive role compared to specialist care, GPs have a higher income than GPs in countries with a weaker primary care sector.

Consequently, this paper will address the following questions:

1. How did the income of GPs develop from 2000 to 2010 in the eight countries in our study (Belgium, Denmark, Finland, France, Germany, the Netherlands, Sweden and the United Kingdom)?

2. Have there been comprehensive changes in the payment systems and did these affect the income of GPs?

3. Is the income level of GPs related to the strength of primary care in these countries?

## Methods

### Operationalizations

We collected annual data on GP-revenues for each country in our study. We distinguish three different revenue components: total revenue, practice costs and income. The total revenue is the amount the GP receives through the remuneration system. For those countries where GPs are self-employed or entrepreneurs, practice costs (consisting of e.g. salary of practice assistants, housing, ICT, transportation and disposables) have to be deducted from the total revenue to calculate income (before taxes). In countries where GPs receive a salary, only data on salaries were collected, since the employer in that case covers practice costs. The income of a GP is presented as the income for a full-time GP after deduction of practice expenses and before income taxes and excludes income from out-of-hours care. The data come from national institutes, most of them are routinely collected data on GP revenues. National data were converted into Purchasing Power Parities US$ (pppUS$) to make the data comparable across countries while allowing for possible differences in purchasing power. The data were then corrected for inflation, with the year 2000 as reference year (Consumer Price Index). Data on pppUS$ conversion rates and inflation were taken from the OECD health data files 2010 [11].

### Comprehensive versus incremental remuneration changes

In this study, we distinguish two types of changes of the remuneration system: comprehensive and incremental changes. We define a comprehensive change as being introduced at a certain point in time (more or less overnight) and changing the main way in which GPs are remunerated (for example from capitation fee to fee-for-service). Incremental changes add new elements to the existing remuneration system or changes the balance between several remuneration types, which are introduced gradually, taking several years to come into full effect (for example, the introduction of a fee for keeping patient’s records, extending the number of patients for which the fee is applicable over the years).

### Strength of primary care

A recent European wide study by Kringos [10] into the strength of primary care distinguished between the primary care structure (using the following dimensions: governance, economic conditions of primary care and primary care workforce development) and the primary care process (access to primary care services, comprehensiveness, continuity and coordination of primary care). On each of the seven dimensions countries were scored from strong to weak on a three point scale. Furthermore a score for total strength of the primary care sector was computed. We will briefly discuss each dimension constructed by Kringos [10]. The governance of primary care refers to the existence of primary care policies and regulations (e.g. on the location of primary care providers and facilities), and the development of the workforce for primary care refers to the workload, age and training of family physicians [12,13]. The accessibility of primary care was measured by the national and geographic supply of primary care services, the way access is organized in primary care practices (e.g. the use of appointment systems, and the organization of out-of-hours care), and the affordability and acceptability of primary care services as perceived by patients. Continuity of primary care was measured by conditions for an enduring doctor-patient relationship (e.g. whether patients are registered with a primary care doctor), provisions to ensure informational continuity of care (e.g. the use of electronic clinical record systems), and elements of the quality of the doctor-patient relationship (e.g. patient perceived available consultation time). Coordination of primary care was measured by the existence of a gatekeeping system, the skill-mix of primary care providers (e.g. % mixed practices with general practitioners and medical specialists; the frequency of face-to-face meetings between general practitioners and other primary care providers), the collaboration within primary care and with secondary care providers (e.g. how common it is for general practitioners to receive clinical lessons by a medical specialist, or for medical specialists to visit a primary care practice to provide joint care with general practitioners), and the integration of certain public health functions in primary care (e.g. whether clinical patient records from primary care are used at regional or local level to identify health needs or priorities for health policy; or whether community health surveys are regularly conducted for public health purposes). The comprehensiveness of primary care was measured by the breadth of services offered to patients at primary care level (e.g. medical technical procedures and certain preventive services). For our study, we could not use the dimension “economic conditions” because GP income was one of the elements of this dimension. The operationalisation of the dimensions and measurement methods have been described by Kringos 2012 [10]. For our study, for each dimension we averaged the income of the countries that scored strong on that dimension and of the countries that scored medium and weak. We then plotted the development in average income over the years for the average income for strong and medium/weak scoring countries. Additional file 1: Table S9 provides an overview of which countries in our study scored strong or medium/weak for each dimension.

### Health care expenditure

Data on health care expenditure were retrieved from the OECD online health database. We related GP income to total health care expenditure on individual health care services (including hospital care, but excluding public health care) and to expenditures on basic medical and diagnostic services, both in pppUS$ per capita. For a detailed description, see the OECD website [14]. In the OECD database, no data were available for the UK on these items.

### Substantial increase in income

For this study, we define a substantial increase in GP income as follows: when the increase is more than the GDP per capita corrected for inflation, we consider the increase as substantial. We look at the increase of GP income in two ways. Firstly, we look at the minimum and the maximum GP income in a certain country in the period under study. Secondly, for countries with comprehensive changes in the remuneration system, we look at the income in the year before the change and the maximum income in the years after the change. The average GDP per capita for the countries included in this study is 29,660 pppUS$ (price level 2000). The minimum average GDP per capita in the past decade is found in France (27,285 pppUS$, price level 2000) and the maximum in The Netherlands (31,472 pppUS$, price level 2000). For a comparison with annual wage development in the population, we used OECD Statistics, i.e. the dataset on average annual wages, in 2009 pppUS$ and 2009 constant prices. The development in annual wages was recalculated using an index with the year 2000 = 100.

### Data sources for GP revenue and practice costs

The countries in our studies can be divided into two groups. The first group consists of the countries that routinely collect data on GP remuneration and practice costs. This is the case in Germany (for public practice only), Finland, France, and the UK. The second group of countries (Belgium, Denmark and the Netherlands) do not routinely collect these data and the income of GPs had to be calculated from other sources, such as public expenditure on GP-care and incidental studies into practice costs. For Belgium, no reliable data source for practice costs was available and thus an estimate was used. For Sweden, where multiple remuneration systems exist, only information on GPs in salaried service was available. The data used for the calculation of total revenue, practice costs and income for each country are available in Additional file 1. Additional file 1 provides the original data per country before correcting for inflation and purchasing power differences, in the original currency of the particular country.

### Data on changes in GP remuneration 2000–2010

Data on changes in the remuneration system for General Practitioners were collected from the Health System Review series published by the European Observatory on Health Systems and Policy and the study of Kroneman *et al* on GP-income from 1975–2005 [8,9]. This information was checked, updated and completed with the help of national experts. National experts were persons working in the field of GP care or payment of GPs either at national governments, national (GP) institutes or universities, depending on the situation of each country and were mainly existing contact persons from our previous study.

Ethical approval was not required for this study.

## Results and discussion

### Changes in the remuneration systems

#### Belgium

GPs in Belgium are mainly paid a fee-for-service. To strengthen primary care, in 1999 the General Medical File (GMF) allowance was introduced for patients of 60 years and older. GPs receive an allowance for all persons for whom they coordinate their medical files. In 2001 the age limit was extended to 50 years and in 2002 the entire population could apply for this GMF coordination [15]. The GMF allowance can be seen as a kind of capitation fee. The share of the capitation fee in GP revenue has increased over time to 11% of the insurance-based revenue (including revenue from out-of-pocket payments) in 2010. Since 2008, also for consultations on weekdays between 18.00-21.00 hours an extra remuneration is received. Several lumpsum payments, such as a lumpsum for accreditation, for settlement (introduced around 2003), a lumpsum for GMF (introduced around 2002), which is additional to the GMF revenue per patient were introduced in the last decade. The GMF lumpsum was replaced by a practice lumpsum in 2008 and a lumpsum for ICT (introduced around 2003). However, no information is available on these lumpsums. In 2009, an integrated care fee for diabetes and chronic kidney disease was introduced. Besides the introduction of the GMF allowance, there have been no comprehensive changes in the remuneration system since 2000. The GMF allowance was introduced gradually over the years. In 2003 there was an increase of 23% in the GMF-tariff per patient and in 2008 the coverage of the GMF increased by 18%. Also in 2008, a supplement of three euro per consultation for out-of-hours consultations was introduced. This resulted in a strong increase in revenue over the years [16], making the remuneration of the GP profession as attractive as other medical specialties (e.g. psychiatrists, paediatricians). An extension of the coverage of health insurance – self employed persons got the same rights as salaried workers in 2008 - resulted in an increase of the official insurance revenues. However, this probably did not increase the total revenues (insurance plus non-insurance) for a GP, assuming that the demand for GP care for self employed persons did not change due to reform. Patients usually pay the GP out-of-pocket and then claim reimbursement from their sickness funds, if applicable [15]. Fee levels for GP-care are negotiated between sickness funds and representatives of the physicians [17].

#### Denmark

In Denmark, the GPs derive their revenues from a combination of a capitation fee, which makes up one third to a half of their revenues, and from fees for services rendered (per consultation, examination, procedure etc.). In the period from 2003 to 2010 there were no significant changes in the remuneration system but only small adjustments in the fees and the types of fees. Priority setting of certain services is introduced in the form of, for example, comparatively high fees for preventive consultations. This higher fee is supposed to encourage GPs to offer longer consultations focusing on broader health and prevention activities such as education regarding smoking or dietary habits, weight control, and so on [18]. In 2006, episode-of-care payment was introduced for patients with diabetes. This fee consisted of a total payment for all consultations given to the patient during one year. It is voluntary for doctors to engage in the program and only a small number of doctors have chosen to do so. GPs receive remuneration for the activities of their practice nurses. We do not have information on whether this revenue covers the costs of these practice nurses.

#### Finland

For general practitioners, who generally work in health centres, two payment systems exist. Traditionally, payment is mainly based on a monthly salary with additional payments based on, among other factors, seniority and skills. Work that exceeds 37 hours a week is remunerated in addition to the salary. The second system is called the personal doctor system, which is a special remuneration formula determined by a basic salary (which can be only about 60–85% of the monthly salary from the traditional system), that is supplemented by a payment per consultation for patients who consult their GP less than three times in the previous year and a monthly “capitation” payment for the so-called frequent visitors - those who visited their GP more than three times in the previous year. This programme leads to higher total revenues compared to the traditional system, but the GP is not protected by the limit of 37 hours per week, as there is no formal working week, just a requirement to offer services on weekdays. Towards the end of the decade, different local modifications of two “official” systems have become more common. Also some municipalities with the personal doctor system have decided to change back to the “traditional” system with doctors having 37 hours per week working time and receiving a salary. Comprehensive changes in the remuneration system have not taken place in the past decade. Another trend during the past 10 years in the municipal sector has been the outsourcing of GP services to private companies. Usually these private companies have their own remuneration system for doctors. Unfortunately current and comparable data on doctors’ salaries employed by these companies are not available.

#### France

General Practitioners in France are mainly paid on a fee- for-service basis. In 2001 supplementary payment was introduced for GPs that registered patients for gate-keeping purposes (“Médecin référent”, referring physician). For each registered patient an extra fee of €46 was introduced [19]. However in 2003, only 1% of the patients and 10% of the GPs joined the programme, and therefore this programme was discontinued. In 2005 the “Médecin traitant” (treating physician) was introduced. This physician manages patients with long-term diseases, receiving €40 per patient per year for this activity. In March 2007, 82% of the insured population had chosen a médecin traitant [20], 99% of whom were GPs. Patients who don’t register with a médecin traitant, or who visit a specialist without consulting their médecin traitant, receive lower reimbursement for the specialist’s fee. The fees for GPs refer to statutory tariffs set out in national agreements. A small part of the GPs (8% in 2008, called ‘sector 2’ physicians) are allowed to charge higher tariffs [21]. However, this system is currently discouraged and only available to physicians with special skills, leading to a further decrease of the percentage of GPs under this regime in the near future. Data on sector 2 physicians are not included in this study.

Since 2009 GPs may receive additional payment for practice improvement. Contracts are signed with the Social Health Insurance on a voluntary basis for a three-year period and can be terminated at any time on the doctor’s demand. The contract encourages GPs to develop prevention, to improve treatment and to follow patients with a range of chronic conditions (currently hypertension and diabetes) and to improve efficiency by increasing the rate of prescription of generic drugs. GPs are offered additional remuneration on top of their normal fee-for-service income. The additional payment takes into account the size of the population treated by the doctor and a number of quality indicators. In 2008, there were 16 indicators for which final but also intermediate targets were defined. Overall, the amount earned can exceed €7000 per year for a doctor achieving over 85% of the targets and treating more than 1200 patients. There is no penalty for GPs who do not achieve the targets.

#### Germany

Until 2008, the payment of ambulatory physicians (both general practitioners and specialists) used to be subject to a global spending cap tagged to the increase of the wage base from which the statutory health insurance funds received income-related contributions. This worked as a two-stage process. First, the statutory health insurance funds made total payments to physicians’ associations in the form of negotiated capitation fees for each member (insured person) of the fund. These negotiated budgets were subsequently distributed among the members of the physicians’ associations according to a uniform relative value scale. This scale contained a list of all services that can be provided by physicians for remuneration within the statutory health insurance system. Each of these services was awarded a certain number of points. Physicians invoiced their associations each quarter for the total number of points generated by the services rendered. The monetary value of the points has been derived by dividing the total negotiated budget (the budget that the GP-unions received from the sickness funds) by total number of points. The monetary value of the points is then used to calculate the physicians’ quarterly remuneration. To prevent physicians from maximising the number of reimbursable points each practice was awarded a budget according to its specialty reflecting within limits historical practice patterns. In 2009 all ambulatory physician services were awarded a Euro-value (based on the previous number of points multiplied by 3.5001 eurocent) thus transforming the uniform relative value scale into a fee schedule. The spending cap was lifted to the extent that physicians’ associations and statutory health insurance funds were collectively required to identify the required budget to cover an expected service level as defined by health risk adjustment (on the basis of age, gender and documented diagnoses for the resident patient population). Prior to this, in 2008, the number of individual services that are applicable for reimbursement was reduced by bundling and the number of capitation-like compensations for a complex of physician services per case (patient/practice/quarter of a year) was increased. Besides, for each GP practice, the maximum number of reimbursable services is settled for each quarter of a year (targets). When a practice produces cases or points above its target, a reduced fee is applicable to all additional services, which are regarded as ‘overproduction’ [22-25].

#### The Netherlands

In 2006 the remuneration system for GPs changed substantially in the Netherlands. Before 2006, two types of patients were distinguished: patients with compulsory public insurance (about 2/3^rd^ of the population) and privately insured patients. For publicly insured patients, GPs received a fixed capitation fee, which was differentiated by age of the patient and whether the patient lived in a deprived area. For privately insured patients, GPs charged fee-for-services (mostly consultations or home visits) to the patients, who charged their health insurance company for full or part reimbursement if GP costs were included in the coverage. Prices were fixed and set by the Dutch Healthcare Authority. After January 1^st^ in 2006, all Dutch inhabitants had to buy the basic package of health insurance, which included GP care. The GPs became remunerated through a mixed system: GPs receive for all patients a capitation fee (which is significantly lower compared to the former capitation fee for publicly insured patients) and a consultation fee that differs for home visits, office consultations and telephone consultations. The capitation fees are higher for older patients and patients living in deprived areas. Furthermore, GPs can negotiate with health insurers for funding of activities that either increase their efficiency or substitute for secondary care. About 8-12% of the GPs revenue is generated via these negotiated activities (own calculations, based on data provided by Vektis).

In the Netherlands, there are self-employed GPs and GPs that are in salaried service of the independent GPs (*huisarts in dienst van een huisarts, HIDHAs*). The share of salaried GPs increased from 7% in 2000 to 12% in 2010 [26]. The income presented in this study is the average income of self-employed GPs.

In 2010 a new type of remuneration was introduced: integrated care remuneration, the so called chain-of-care DRG (*keten-DBC*) for diabetes, COPD and CVA, consisting of a lump-sum payment for each patient with one of these diseases in the practice. With this money, all care of these patients has to be paid, also care provided by secondary health providers (hospitals and specialists). Converted to an FTE GP, this results in an extra remuneration of 16.800 euro (about 6% of the total revenue). At present, it is unclear how this money is spent in practice, and thus it is unknown what part is GP income and which part consists of additional costs.

#### Sweden

In Sweden, county councils are responsible for ambulatory health care provision. Payments to primary care centres are normally based on all-in budgets, and payment principles may vary between the county councils. Physicians are in salaried service at primary care centres. As such, they receive a monthly salary from the county council. In some counties GPs receive an additional capitation fee for each patient, to increase their monthly income. In the last decades, the differentiation among the Swedish counties has increased and nowadays there are as many health care systems as there are counties (21) in Sweden, with all counties operating with a different mix of salary, capitation and fee-for-service for GPs, which makes it impossible to determine “the” Swedish GP remuneration system. In the period from 2000 to 2010, no major changes have taken place, at least not at national level.

#### United Kingdom (England)

Before 2004, the revenues of GPs in the United Kingdom were determined by a basic allowance, supplemented by allowances based on factors including the number of listed patients, patient characteristics (age, chronic conditions, living in deprived areas) and some types of services rendered. There was a slight gradient in basic allowance due to seniority (depending on the number of years a GP is registered).

In 2004, the payment system changed from GP-based to practice-based, with an all-in budget (the global sum). Payment was still based on characteristics of the practice”s patient list, but additional revenues could be earned when certain quality requirements were met (specified in the quality and outcomes framework (QOF)). For this performance payment, four domains for quality improvement were introduced: the clinical domain (with an emphasis on certain diseases), the organizational domain (including: information, communication, education and practice management), the additional services domain (cervical screening, child health surveillance, maternity services and contraceptive services) and finally the patient experience domain which consists of how services are provided and the involvement of patients in service development plans. The UK is the first country in Europe to use patient surveys to reward GP-practices. Revenues generated from the QOF may increase total practice revenue by 15-20% [27]. In the first two years of the contract, a total of 1050 points per year could be earned, with a value of £76 per point in the first year and £125 per point in the second year. The UK practices achieved over 90% of these available points [27,28]. In the third year the number of points to be earned dropped to 1000 [29]. In 2012/13 the value per point was £134 [30]. Besides the global sum and the quality and outcomes framework, practices may receive remuneration for additional services that they may choose to provide, the directly enhanced services. Examples are screening for hazardous alcohol consumption and extended practice opening hours (three hours of extra appointment time per day).

There are two different contracts for GP-practices, the General Medical Services contract (GMS) and the Personal Medical Services contract (PMS). The GMS is negotiated between the British Medical Association and the NHS at the national level. The PMS is negotiated between local level health authorities (in particular, Primary Care Trusts) and individual GP-practices. The income of PMS contracted GPs in 2008 is on average higher compared to GMS-contracted GPs (about 16%, calculation based on data from the Earnings and Expenses Report 2007/2008 [31]). The share of PMS contracted GPs has increased from about 4% in 2000 to 52% in 2010 [32,33]. Besides the contractor GPs, there is a growing number of GPs who are working in salaried service. These GPs earn on average £55,800 (87,000 pppUS$) in 2007–08 per FTE. Most of them work part time (on average about 24 hours per week) [34].

### Changes in paying the GP

Countries with a single remuneration system to pay the GP do not exist anymore in 2010. Remuneration systems for GPs have increased in complexity and detail. Even in countries where (most) GPs are in salaried service, experiments with additional payments in the form of, for example, fee-for-service have been introduced. New forms of payment that have been introduced in the past decade are the integrated care fees and the performance fees. The integrated care fee refers to a system where GPs receive an amount per patient with a certain diagnosis (for example diabetes) and this amount should be used to cover all health care costs of the specific patient, including hospital and specialist care. This is a new development, which does not (yet) form a significant share of total revenue. Integrated care fees have been introduced in Belgium, Denmark, Germany, and The Netherlands (see Table 1). Another innovation is the introduction of” performance fees’, payments that are payable after a certain target is reached. For instance, when a predefined percentage of the high-risk population is vaccinated against influenza, the GP receives a certain payment. Performance fees have been introduced in France and the UK (see Table 1).

**Table 1:** **Main types of remuneration of GPs in eight countries in 2010 with *new *** **(italic) and changed (underlined) remuneration types compared to 2000**

	**Salary**	**Fee-for-service**	Capita-tion fee*	**Perfor-mance fee**	**Integrated care fee**	**Other**	**Type of change**
**Belgium**		Yes	*Yes*		*Yes*		Incremental
**Denmark**		Yes	Yes		Yes**		Incremental
**Finland**	Yes	Yes	Yes				Incremental
**France**		Yes	*Yes*	*Yes*			Incremental
**Germany**		**Yes**			Yes**		Comprehensive
**The Netherlands**	***	**Yes**	**Yes**		Yes**	*Yes*	Comprehensive
**Sweden**	Yes						Incremental
**UK (England)**	***		**Yes**	*Yes*			Comprehensive

If the text “Yes” is in *italic*, this type of remuneration is new for this country and if the text is underlined, this type of remuneration has changed in that particular country in 2010 compared to the year 2000.

* Capitation fee here includes also the fees for keeping a patient’s record.

**The integrated care fee is fairly new and in none of the countries it forms a significant share of the total revenue.

*** In the Netherlands 7-12% of the GPs are in salaried employment with independent GPs in the past decade. In the UK, also a growing number of GPs in working in salaried service, from 10% in 2004 to 19% in 2008 (including GPs working in salaried service and GPs who work flexible arrangements, excluding trainees (GP registrars) [34].

In the past decade, in the UK (in 2004), the Netherlands (in 2006) and in Germany (in 2008), comprehensive changes in the GP-remuneration system took place. In the UK, the payment became practice based and performance payments were introduced that formed a substantial part of the practice income. In the Netherlands the difference between the payment systems for publicly and privately insured people was abolished and merged into one payment system for all citizens. For GPs it was difficult to predict what would be the income under the new system beforehand, since in the former system for the previously publicly insured population no fee-for-service existed and the existing administrative systems of the GPs did not provide insight into the income generated in the new system. In Germany, the budget cap was released, and although there are reduced tariffs above a certain number of reimbursable services, there is no limit in the number of reimbursable services anymore. In the other countries in our study incremental changes took place, varying from an increase in tariffs for certain remuneration elements to changes in the share of the different remuneration elements in the total revenue and the introduction of new remuneration elements in addition to existing elements, such as remuneration for keeping patient’s records. This was the case in Belgium and France.

### Income development in the eight countries

**Figure 1:**
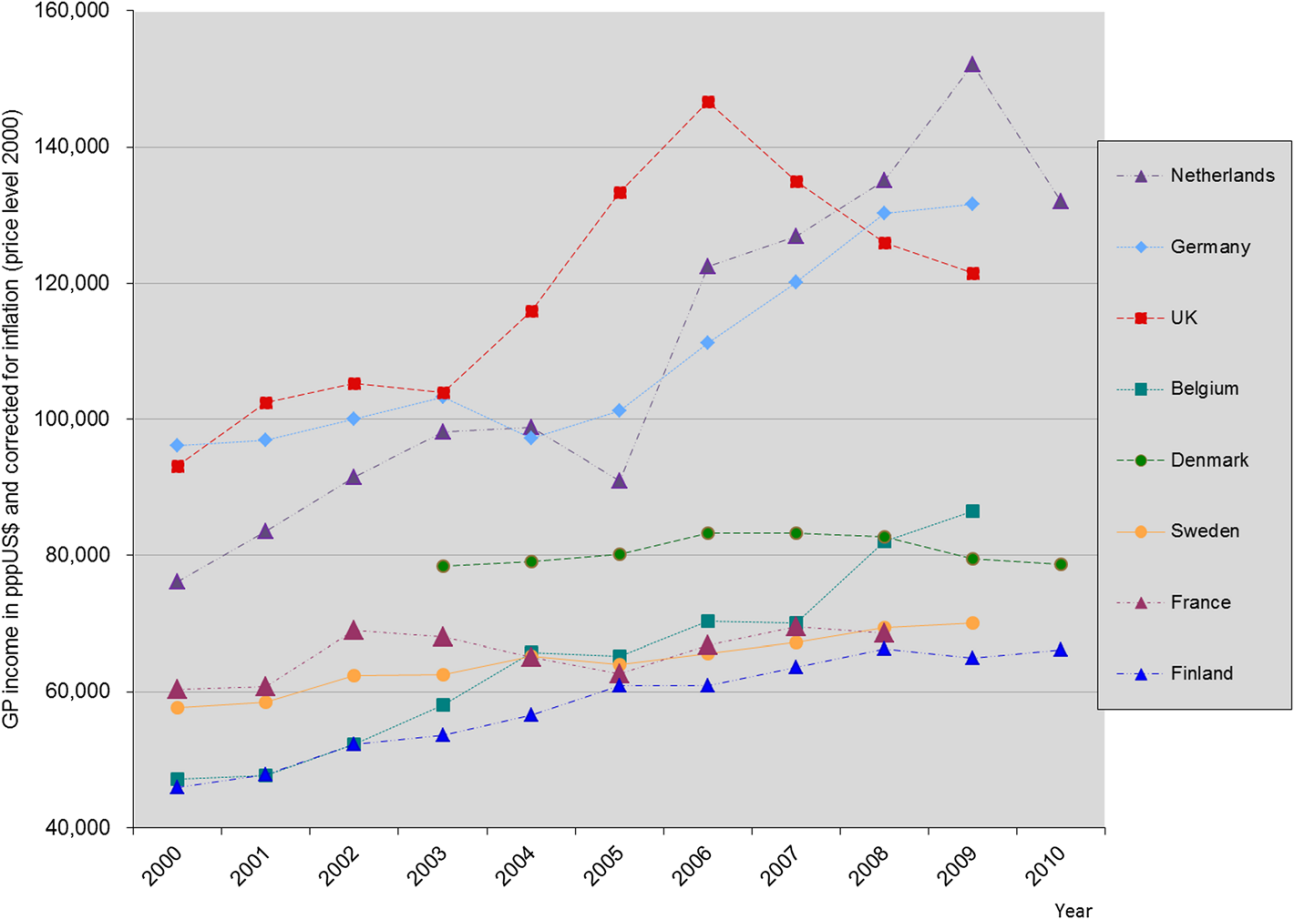
GP-income development over time in pppUS$ and corrected for inflation (price level: 2000).

**Figure 2:**
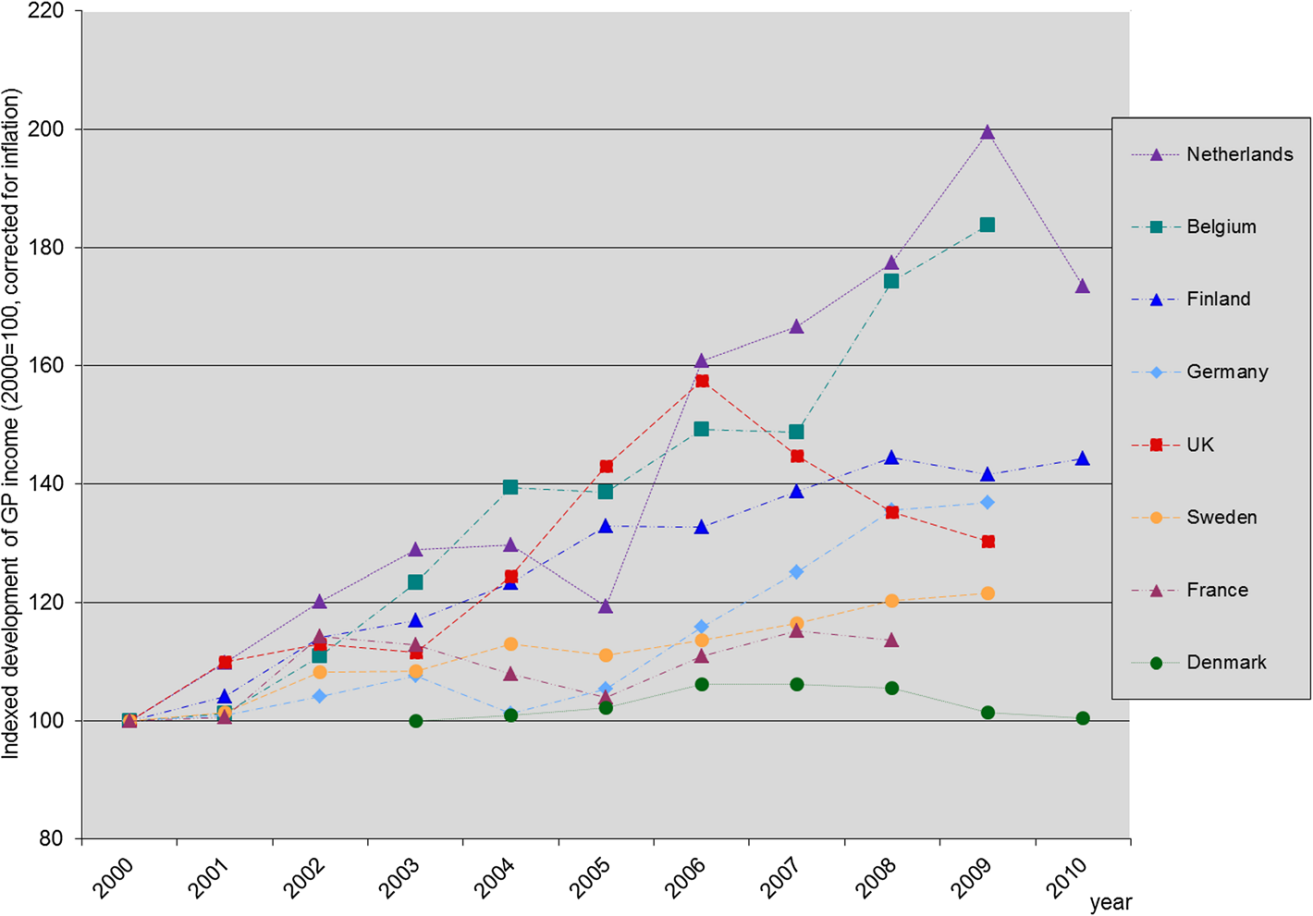
Relative income development of GPs (2000 = 100; for Denmark: 2003 = 100).

In Figures 1 and 2, the income of GPs from 2000 to 2010 (or latest available year) is displayed (in pppUS$, corrected for inflation and indexed with 2000 = 100). Two of the countries with major changes in the remuneration system (the Netherlands and the UK) show a substantial increase in income immediately after the reform and the subsequent two to three years. The total increase exceeds the average growth in GDP per capita. However, after about three years, the evolution of income is decreasing again in both countries (although for the Netherlands, only one year is available with a lowered income, so it is not clear whether this is a continuous trend). In Germany, where the change was introduced in 2008, in the next year an increase was observed, but since the change was introduced only recently, no data were available for the next years, so here it is also not clear whether this is a trend. The increase between 2008 and 2009 was less than the average growth in GDP per capita. In the countries with incremental changes, much lower increases were found. In Denmark, Finland, France, and Sweden, the increase was less than the average growth in GDP per capita. Only for Belgium, the increase was substantial (more than one time the average growth in GDP per capita).

### Income development and the strength of primary care

**Figure 3:**
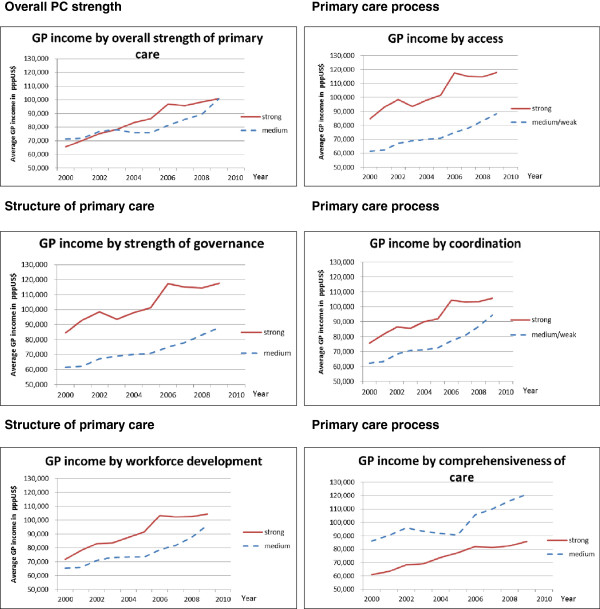
Average GP income of countries scoring strong or medium/weak on dimension of primary care strength*. * Countries scoring strong on the dimensions of primary care (included in calculating the red lines: Total strength: Belgium, Denmark, Finland, Netherlands, UK. Governance: Denmark, Netherlands, UK. Workforce development: Denmark, Finland, Netherlands, UK. Primary care access: Denmark, Netherlands, UK. Coordination of care: Denmark, Netherlands, Sweden, UK. Continuity of care: Denmark, Belgium, Germany. Comprehensiveness: Belgium, Finland, France, Sweden, UK. The countries that are not mentioned here scored medium or weak on the different dimensions and are included in calculating the blue lines.

The total strength of the primary care sector was not related to differences in GP income (Figure 3). When we consider the key dimensions of primary care strength a more clear association between primary care strength and income development appeared. GPs working in countries with strong primary care governance, a strongly developed primary care workforce, accessible and well coordinated care have a continuously higher income level than GPs working in countries with a medium or weak primary care sector. The difference over time in income of GPs working in countries with strong and medium PC seemed to be rather stable. The increase in income seemed to be roughly similar in both types of primary care systems. Overall, there appears to be no relationship between the income level of GPs and the continuity of care provided by primary care physicians. The comprehensiveness of primary care services showed a striking relation with income: GP income in countries with comprehensive primary care was on average lower compared to countries with a less comprehensive primary care system (see Figure 3).

### Health care expenditure and GP income levels

**Figure 4:**
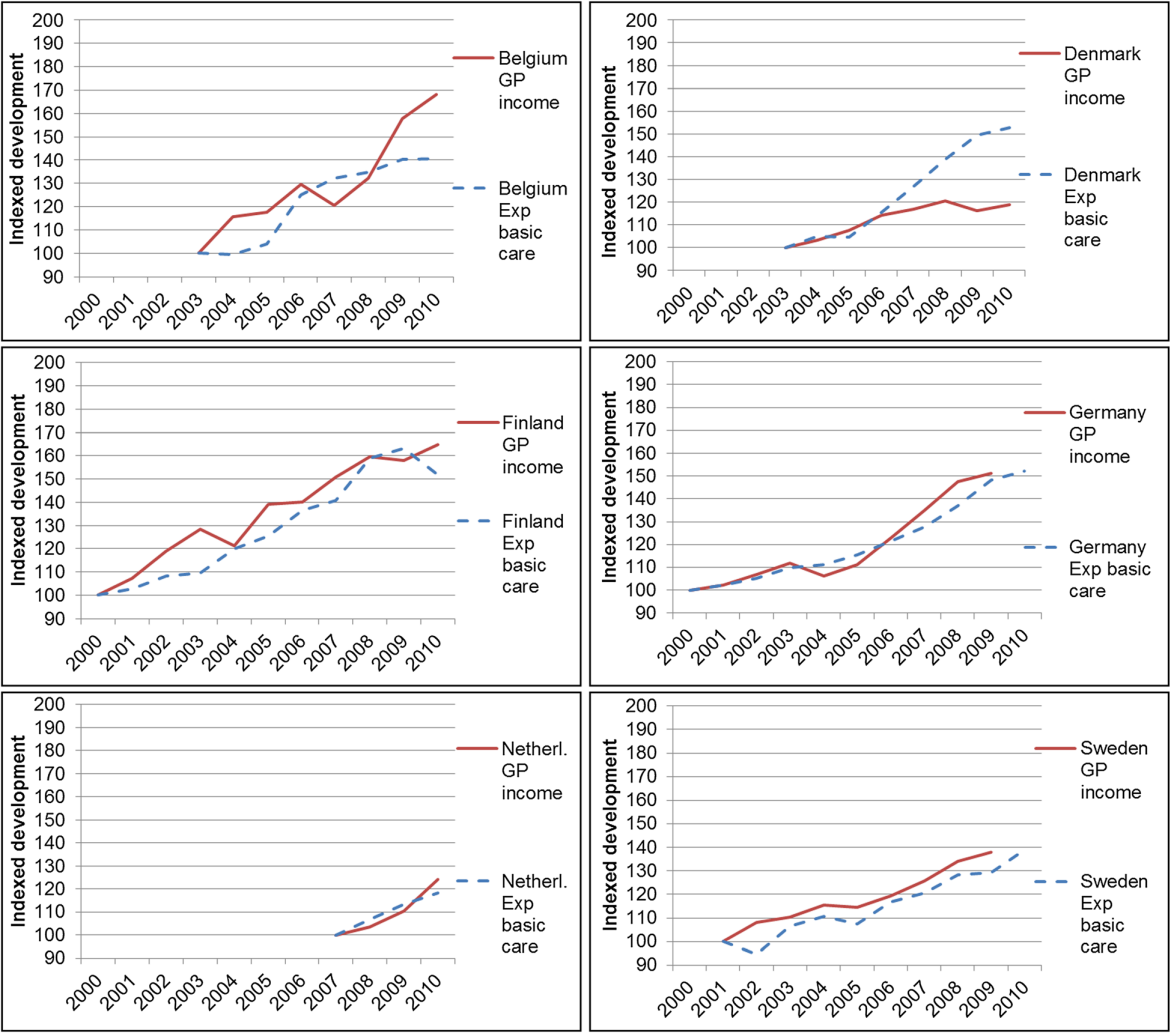
**Relative income development compared to relative development in expenditure in basic medical and diagnostic care (OECD-data) per country, 100 = first year of data available in both time series.** Exp. basic care: Expenditure in basic medical and diagnostic care. No data on expenditure in basic medical and diagnostic care available for France and UK.

In 2009 (for France: 2008) GP income was moderately correlated to expenditure on individual health care services (pearson correlation coefficient = 0.60). Surprisingly, GP income was negatively correlated to expenditure on basic medical and diagnostic services (pearson correlation coefficient = −0.45). The development in GP income over the decade was strongly correlated with the development in expenditure on basic medical and diagnostic services, with correlation coefficients varying between 0.78 for Sweden to 0.98 for Germany. There were two outliers: The Netherlands (0.58) and France (0.32). For the Netherlands, the length of the time series was only four years. For France, there was a break in series that made comparison over time invalid. When looking at the development as index (2000 = 100), we see that in Belgium the GP income increased more compared to basic medical care expenditure and that in Denmark the expenditure increased stronger than GP income (Figure 4). For the other countries the increase was more or less the same.

### Income development of GPs in relation to GDP and average income of the population

For all countries, in monetary terms corrected for inflation, the largest increase in income between 2000 and 2009 took place in the Netherlands (more than two times the average GDP per capita), followed by Belgium, Germany and the UK, respectively (more than one time the average GDP per capita). In Sweden, France and Denmark the lowest increase was found. The relative change was highest in the Netherlands and lowest in Denmark (see Figure 4). If we compare this with the growth in GDP per capita corrected for inflation from 2000 to 2009 (which is between 3 and 15% in the countries in this study), we see that for all countries except Denmark, the GP income has increased more than the GDP per capita. When we compare the income development of the total population from 2000 to 2009 with GP-income development, we see that income of GPs in Belgium, Germany and the Netherlands have increased considerably more than the income of the average population (more than 30%), the income of GPs in Finland and the UK increased around 20% more than the average income of the total population, and the income of GPs in France and Sweden have developed more or less similar to the income of the average population. The income development of GPs in Denmark remained below the development of the average income in the general population (−14%).

### Practice costs

The share of practice costs in total revenue is rather similar between countries, independent of the level of income. The share is around 50% (varying from 46% in France to 57% in the UK). Belgium formed an outlier: the share here is 35%. However, the estimate of the practice costs for GPs in Belgium was not based on survey or tax-based data, resulting in a less reliable estimate.

## Discussion

When we look at the development of the income of the GPs between 2000 and 2010, several conclusions can be drawn. Firstly, the incomes of GPs in all countries except Denmark have increased (corrected for inflation), and increased more than the average income in the population. The largest relative increase was found in the Netherlands and Belgium. Secondly, the gap between the countries where GPs have a relatively lower income (Belgium, Sweden, France and Finland) and the countries where GPs have a higher income (Netherlands, Germany and the UK) continues to exist over time. Thirdly, the countries with comprehensive reforms are the countries where GPs received the highest payments already. The result of the comprehensive change seems to be that GP income increased substantially in the two or three years after the change. After that, the income decreased somewhat. In the UK, an increase in income was intended [27,28], but the increase was higher than foreseen [27]. In Belgium, the increase in income was also intended to make the profession more attractive in comparison with other medical specialties [16]. In the Netherlands, the change was intended to be budget neutral, as in Germany.

Fourthly, on average, GPs working in countries with a strong primary care sector in terms of governance, workforce development, access, and coordination of care appear to have a substantial higher income than GPs working in countries with a medium or weak primary care sector. We found no association between income development and the continuity of primary care. Perhaps this can be explained by the fact that having a long-term relationship with patients, keeping medical records, and creating a feeling of trust is a universal feature of primary care and inherent to the profession of GPs, irrespective of their income level, while governance, workforce development, access, and coordination of care are largely influenced by policies and financial incentives. It is unclear why GPs in countries with a comprehensive service delivery earn less compared to their colleagues in countries with a less comprehensive service delivery. The dimension of comprehensiveness was mainly based on expert opinion, whereas the other dimensions are based on more robust data [10]. Maybe experts tend to overestimate the comprehensiveness in their country. All in all, the results seem to suggest that in countries with a better organized primary health care system, GPs have a relatively stronger position in the system and are also able to use this position to their own benefit, i.e. by ensuring a higher income.

Policy makers have tried to curb the unintended increases in income in the UK and the Netherlands. In the UK, the GMS contract of 2004 was valid for three years. So the first year of possible measures to curb the increase in income was 2007. In the following years, net remuneration of GPs was either frozen or increased with only a (partial) compensation for inflation [27,33]. The quality and outcomes framework was adjusted several times. The idea behind the changes was to continuously improve the quality of the general practice. The effect was that income of GPs in real terms decreased after the initial GMS contract had ended, but remained higher than the level before the reform. In the Netherlands, where a budget neural transition was envisaged, the unforeseen increase led to a claim on GPs to repay the excess, which is currently under discussion between representatives of the GPs and the Ministry of Health.

### Limitations of the study

An important limitation of this study is that we only present average incomes of GPs. The variation in income within countries in the case of non-salaried GPs are large. Evidence for this is found in Belgium [35], the Netherlands [36], Germany [37] and the UK [38].

A problem in calculating GP income from the total revenue is the increasing complexity of the financing of GP practices. The emergence of new payment systems, such as the integrated care fees and new health professionals working for GPs, such as nurse practitioners, make it difficult to establish the expenditure of GPs, since for instance health care consumption of specialist care of patients on the GP’s list have to be paid by the GP(−practice). There is no longer a relationship between workload (in number of patients or number of services) and remuneration. This will make it difficult to monitor GP-expenditure in the future.

We have tried to relate the development in GP income to the context of primary care. However, we are aware of the fact that we only have eight countries in our study. Therefore, the results should be seen as a first indication of the association between income development and primary care context.

Further limitations concern the calculation of practice costs, the method of calculating GP income before and after a drastic reform, the information on number of active (fulltime) GPs, and the income derived from services for private patients.

The most important problems in calculating GP income in countries where GPs are self-employed entrepreneurs are to calculate practice costs and to define what is a full-time GP. Practice costs include salaries for practice personnel, housing, medical equipment, transportation etc.. The most reliable data on practice costs come from tax data. However, this implies that the tax office knows who are GPs and how many GPs are actively working as GP. Since this information is not always available, in some countries dedicated studies have been carried out into this matter (as for instance in Denmark, the Netherlands and Germany [39-41]. These studies are usually not repeated on a yearly basis, thus estimation methods have to be used to calculate practice costs for the years where no data were available. Besides, the outcome of the studies into practice costs are related to the organization that initiates the study. Parties that represent the interest of GPs often have a systematically higher estimate compared to paying parties, such as governments and health insurers [8]. The definition of what is a full-time GP may change over time, leading to different income figures. In Belgium, for instance, a continuous effort to improve the estimation of the GP’s context lead to different methods of calculation and thus to different figures of GP income.

For the comparisons within countries, as a result of changing remuneration methods, the calculation of the income may contain different income components before and after the reform. In the Netherlands, for instance, this may underestimate the income of GPs before 2006, since GPs may receive some extra remuneration through extra allowance for practice management and from extra activities such as pap smears, health checks for drivers’ licences, making an ECG etc. These income components (besides influenza vaccination) are not included in the calculations. However, the underestimation will not be so large, that the difference between 2005 and 2006 can be explained from this artefact. For the comparison between countries, we have to note that there is an underestimation of the income of GPs in Denmark, because the income is based on head counts of GPs in stead of full-time equivalents. There is a difference of around 20% for the three years that data are available for both full-time GP and income based on head count. However, since the number of GPs has not changed substantially over the years, we assume that the underestimation is constant over time. In the data for Belgium, Denmark and France, income from out-of-hours activities are included, whereas this is excluded in the other countries, leading to a slight overestimation of the income of Belgium, Danish and French GPs compared to the other countries.

Finally, changes in GP remuneration may not be the only cause of changes in income. Other developments, such as increasing demand for GP services, ageing of the population and substitution of hospital care to primary care may also affect GP income. In the Netherlands, for instance, there is evidence that part of the increase is due to an increase in primary care use [42].

## Conclusions

Summarizing, we found that comprehensive changes in the remuneration system of GPs may influence GP income considerably. Incremental changes are associated with a gradual increase in income after correction for inflation, with the exception of Belgium where the incremental change coincided with a considerable increase. Beside the traditional payment systems, i.e. salary, capitation fee, and fee-for-service, new payment systems have been introduced to promote and reward quality and integration of care through financial incentives. Examples are pay-for-performance, which is most elaborated in the UK, and integrated care payment systems. The latter is still in development and is facing problems due to the differences in payment systems of the different health care providers [7]. In countries with a strong primary structure, GP income was on average higher, especially for countries with a strong primary care governance. For the primary care dimensions on service delivery, a strong access and coordination of care were associated with a high GP income, whereas a strong comprehensiveness and continuity of services were associated with a lower GP income. We do not have an explanation for this latter finding. In all countries, except Denmark, GP income increased more than average wages. For the comparison of income between countries, we have tried to harmonize the type of income elements included in the calculations as much as possible, but the available data were not always straightforwardly comparable. Thus, the figures presented in this study should be interpreted with caution. New payment forms, such as integrated care payment systems, and new health care professionals that are working for GPs, increasingly blur the line between practice costs and income, making it more and more important to define clearly the expenditure of GPs, to remain sight on the actual income of GPs.

## Abbreviations

DRG: Diagnosis related groups; COPD: Chronic obstructive pulmonary disease; CVA: Cerebrovascular accident; ECG: Electrocardiography; FTE: Full time equivalents; GMF: General medical file; GMS: General medical services contract; GP: General practitioner; NHS: National health service; OECD: Organisation for economic co-operation and Development; PMS: Personal medical services contract; US$: United States dollars in purchasing power parities; QOF: Quality and outcomes framework; UK: United Kingdom (in our case: England).

## Competing interests

All authors declare to have no competing interest: no support from any organisation for the submitted work; no financial relationships with any organisations that might have an interest in the submitted work in the previous 3 years; no other relationships or activities that could appear to have influenced the submitted work.

## Authors’ contributions

MK drafted the manuscript, carried out the analyses and collected the data. PM carried out the calculations and analyses for Belgium. DSK provided the data on primary care dimensions and helped with the interpretation and helped to draft the manuscript. WG conducted a critical review of the manuscript and helped with the analyses. JZ initiated the study, participated in the design and helped to draft the manuscript. All authors read and approved the final manuscript.

## Pre-publication history

The pre-publication history for this paper can be accessed here:

http://www.biomedcentral.com/1472-6963/13/436/prepub

## Supplementary Material

Additional file 1 Calculation of GP income. Click here for file
