# Supplementary material for: International developments in revenues and incomes of general practitioners from 2000 to 2010
Source: BMC Health Serv Res. 2013 Oct 24;13:436. doi: 10.1186/1472-6963-13-436 (PMC4015771; doi:10.1186/1472-6963-13-436)
Supplement: Additional file 1 — Calculation of GP income. [file 1472-6963-13-436-S1.doc]

# Additional file 1: Calculation of GP income

In this Additional file, the sources and, where applicable, the calculations of the GP income in the countries under study have been described. The data are provided in the local currencies. Each country description ends with an evaluation of the peculiarities of the data compared to the real situation in that country. After the country descriptions, the figures comparing the income of GPs with the score on primary care strength dimensions are given, together with an overview of the scores (weak, medium, strong) of each country on the dimensions and the figures comparing the growth in GP income with the growth in expenditure on basic medical care in each country. At the end of the Annex, the conversion rates for conversion into pppUS$ and the consumer price indices used for the inflation correction have been displayed.

*Belgium*

Data source: RIZIV, the National Institute for Health and Disability Insurance provided the data for the insurance based revenue of fulltime GPs.

Calculation: For 2000-2005, a full-time GP in Belgium was defined as working approximately 41-42 hours per week, taking care of on average 802 patients (588-1056) and having on average 5023 patient contacts (3651-6962) per year. About 45% of all GPs work fulltime[1] according to this definition. From 2006 onward, a full-time GP is defined as a GP earning the median revenue of a GP aged between 45 and 54 year of age. The total revenues of GPs include remuneration for out-of-hours services. For 2006 to 2010, also data on the revenue from private expenditures of patients was available. For the years before 2006, we estimated the income generated from private expenditures by using the percentage on private expenditures for the year 2006. Routinely registered data on practice costs of GPs were not available, nor are there specific studies into the practice costs. An estimation of practice costs was made by the ASGB (Algemeen Syndicaat van Geneeskundigen van België; Association of Belgian Physicians) in 2001 in the form of a discussion paper. Since this was the only written source on practice costs, we used these figures to calculate practice costs for other years as well, after correction for inflation [2].

Table S1: GP income in Belgium in Euro

| **year** | **Average income full-time GP, insurance based** | **Additional income from private contributions (%)*** | **Total revenues** | **Practice costs **** |
| --- | --- | --- | --- | --- |
| 2000 | 66.974 | 21,85% | 81.608 | 41.099,90 |
| 2001 | 68.979 | 21,85% | 84.051 | 41.963,00 |
| 2002 | 71.780 | 21,85% | 87.464 | 42.785,00 |
| 2003 | 79.334 | 21,85% | 96.668 | 43.648,10 |
| 2004 | 86.434 | 21,85% | 105.320 | 44.593,39 |
| 2005 | 91.037 | 21,85% | 110.929 | 45.661,99 |
| 2006 | 97.046 | 21,85% | 118.251 | 46.689,49 |
| 2007 | 101.321 | 19,81% | 121.393 | 47.799,19 |
| 2008 | 113.115 | 20,23% | 135.998 | 48.703,38 |
| 2009 | 117.462 | 21,30% | 142.481 | 49.237,68 |

*Private out-of-pocket contributions from patients. For the years 2000-2005 no data were available and we used the same percentage as in 2006.

**Source: ASGB ontbijtpaper 2002[2], the other years are based on the figure for 2001, corrected for inflation.

Peculiarities: The data do not include several lump sum payments such as lump sums for accreditation, settlement, General Medical File (GMF) lump sum (which is additional to the GMF revenue per patient), and ICT; these data were not available to us. The data include supplement fees for services and activities that are produced in weekends. The productivity of GPs has increased over time: GPs tend to have larger practices, with more consultations, but with less home visits. In 2000, on average, a GP had 800 patients, in 2009 this increased to 1003 patients [3].

*Denmark*

Data sources: Data on total revenues came from Danske Regioner (Danish Regions), the organisation that represents the interest of the five regions in Denmark. Practice cost data were available for 2004, 2006 and 2009, together with the share of practice costs in total revenues from a study of the PLO (Praktiserende Lægers Organisation, the Danish GP trade union) [4].

Calculations: Danske Regioner had information on total public revenue of GPs. Revenue from private practice is unknown, but according to Danske Regioner, the contribution of private practice to total revenue is relatively small. Practice cost data were available for 2004, 2006 and 2009, together with the share of practice costs in total revenues from a study of the PLO (Praktiserende Lægers Organisation, the Danish GP trade union) [4]. Since the total revenue data from the PLO study did not match the data from Danske Regioner, the PLO data came from a sample (550 GPs, about 15% of the population), Danske Regioner provided data on the total population of GPs and Danske Regioner had data for more years available, we decided to use the share in practice costs from the PLO study and thus estimated the practice costs by calculating the total revenue available from the Danske Regioner times the share of practice costs in total revenues as calculated by PLO. For the years 2005, 2007 and 2008 the share of practice costs was estimated using a moving average method. For the years 2003 and 2010, the same share as the following and previous year was used, respectively.

Peculiarities: For Denmark, the figures are based on the number of GPs, since data on full-time equivalents of GPs were not available. Thus these figures may represent an underestimation of the income of a full-time working GP. However, data provided to us by PLO revealed that the average working time of GPs in 2006 en 2009 was around 45 hours per week, which may be considered full-time. Contrary to our definition of GP-revenue, the total revenue from Danske Regioner included remuneration for out-of-hours activities, which may lead to an overestimation of GP-income.

Table S2. GP remuneration in Danske Kroner

|  | **Revenue** | **Practice costs** | **Income** | **Share of practice costs** | |
| --- | --- | --- | --- | --- | --- |
| **Year** |  | **= Share of practice costs * Revenue** | **= Revenue - practice costs** |
| **2003** | 1,373,698 | 654,459 | 719,239 | 47.6% | * |
| **2004** | 1,403,375 | 668,598 | 734,777 | 47.6% |  |
| **2005** | 1,439,982 | 666,987 | 772,996 | 46.3% | ** |
| **2006** | 1,506,879 | 678,036 | 828,844 | 45.0% |  |
| **2007** | 1,564,840 | 737,524 | 827,316 | 47.1% | *** |
| **2008** | 1,666,660 | 821,095 | 845,565 | 49.3% | *** |
| **2009** | 1,697,445 | 872,501 | 824,945 | 51.4% |  |
| **2010** | 1,736,075 | 892,357 | 843,719 | 51.4% | **** |

*Practice cost share based on 2004

**Practice cost share based on average between 2004 and 2006

***Practice cost share based on moving average between 2006 and 2009

****Practice cost share based on 2009

*Finland*

Data source: Statistics Finland, data provided by the Finnish Medical Network (FiMnet).

Calculations: the data provided were average salaries of GPs.

Peculiarities: The dip in income in 2009 is probable due to data collection problems and does not represent a real dip.

Table S3. GP income in Finland in Euro

| **Year** | **Day time earnings, in euros per month** | **Annual income per year** |
| --- | --- | --- |
| 2000 | 3.702 | 46.275 |
| 2001 | 4.065 | 50.813 |
| 2002 | 4.333 | 54.163 |
| 2003 | 4.549 | 56.863 |
| 2004 | 4.687 | 58.588 |
| 2005 | 4.989 | 62.363 |
| 2006 | 5.097 | 63.713 |
| 2007 | 5.325 | 66.563 |
| 2008 | 5.559 | 69.488 |
| 2009* | 5.515 | 68.938 |
| 2010 | 5.717 | 71.463 |

* For 2009 the data are incomplete, there were some methodological difficulties in data formation (part of the capitation fees were missing, for instance).

*France*

Data source: publications from DREES, Direction de la Recherche, des Études, de l’Évaluation et des Statistiques, the department of research, studies, evaluations and statistics of the Ministry of Labour and Health [5-8].

Peculiarities: The French data on GP-income were available only after deduction of social security contributions, which may lead to an underestimation of the income of the French GP compared to the other countries. The data on GP income are published after deduction of practice costs.

Table S4. GP income in France in Euro

| **Year** | **Income** |  |
| --- | --- | --- |
| 2000 | 53.400 | * |
| 2001 | 53.614 | ** |
| 2002 | 59.731 | ** |
| 2003 | 64.000 | ** |
| 2004 | 61.805 | *** |
| 2005 | 63.900 | * |
| 2006 | 67.350 | **** |
| 2007 | 70.940 | **** |
| 2008 | 71.690 | **** |

*Kroneman e.a., 2009[9]

**Legendre, 2005, no 412[8]

***Legendre, 2007, no 562[6]

****Bellamy, 2010[5]

*Germany*

Data source: Data for the remuneration for the treatment of publicly insured patients (89-90 % of the population in the past decade (OECD health data files, online, access 13 jan 2012)) are published by the Kassenärztliche Bundesvereinigung [10-17]. The data for 2006-2008 were derived from the Zi-Praxix-Panel, Jahresbericht 2010[18].

Table S5. GP income for Germany in Euro

| **Year** | **Revenue from insurance based activities** | **Revenue: Insurance + private (17%)** | **Share of practice costs** | **Practice costs**  **= Revenue insurance based * share of practice costs** | **Income** |
| --- | --- | --- | --- | --- | --- |
| 2000 | 168,624 | 197,290 | 55.0% | 108,510 | 88,781 |
| 2001 | 171,700 | 200,889 | 55.0% | 110,489 | 90,400 |
| 2002 | 173,200 | 202,644 | 55.0% | 111,454 | 91,190 |
| 2003 | 179,400 | 209,898 | 55.0% | 115,444 | 94,454 |
| 2004 | 166,900 | 195,273 | 55.0% | 107,400 | 87,873 |
| 2005 | 175,900 | 205,803 | 55.0% | 113,192 | 92,611 |
| 2006 | 178,600 | 211,892 | 53.8% | 113,934 | 97,958 |
| 2007 | 187,400 | 221,959 | 52.1% | 115,713 | 106,246 |
| 2008 | 184,800 | 230,971 | 50.4% | 116,307 | 114,664 |
| 2009 | 206,368 | 241,451 | 51.7% | 124,830 | 116,621 |

Calculations: For 2000-2004 we used the remuneration published for the Allgemein Ärzte (literally: general practitioners). For 2005-2009 the wording changed in Hausärzte (literally: home practitioners). The revenues generated by treating private patients was derived from the Zentralinstitut für die kassenärztliche Versorgung in Deutschland (Zi), the National Institute for GP-care in Germany and based on data for 2000 and 2005 [19]. For the years where no data were available, we extrapolated the private revenues from other years and corrected for inflation. The share of practice costs was available for 2000-2003 (55%) and 2009 (51.7%). We calculated the practice costs in the years in between by using a percentage of 55%, to avoid overestimation of GP-income.

Peculiarities: Income from private sources was limited available (only for 2000 and 2005).

*Netherlands*

Data sources: In the Netherlands, data on GP remuneration and practice costs are not routinely collected. Therefore, the income of a Dutch full-time GP had to be calculated using data from different sources. For the period 2000-2005 the following sources were used: Data on the number of GPs, and for the years 2001 on GP productivity (number of consultations for private patients and number of publicly insured patients) were collected in a large study into GP care conducted in 2001 by NIVEL, the Netherlands Institute for Health Services Research [20-26]. Tariffs were derived from the yearly publications of the successive bodies that settled GP tariffs (the Board for Health Care Tariffs and since 2006 the Dutch Healthcare Authority) [27-32]. For the period 2006-2010 due the change in the organisation of the health care in the Netherlands, different sources had to be used, probably leading to a break in series. For expenditure on General Practice care in 2006 and later, data from Vektis, the Information Centre for Healthcare established by Dutch health care insurers, were used [33]. GP-practice costs for 2006 and later are based on a study of Significant, an independent consultancy agency[34], that performed a study commissioned by the Dutch Healthcare Authority for the years 2009 and 2010 in combination with the figures on GP practice costs published by Statistics Netherlands [35]. Data on influenza vaccination uptake were derived from the national influenza vaccination monitor [36-38] and tariffs for the subsequent years were provided by the National Foundation for Influenza Vaccination.

Calculations: Revenues for the years 2000-2005 were calculated through productivity data of NIVEL in 2001 and by tariffs derived from the yearly publications of the successive bodies that settled GP tariffs (the Board for Health Care Tariffs and since 2006 the Dutch Healthcare Authority) [27-32], assuming that there were no changes in productivity between 2000 and 2005. GP-practice costs for 2000-2005 are based on the study of Statistics Netherlands in 2001 and extrapolated to the other years, corrected for inflation. Revenues for the years 2006-2010 were calculated as follows:

The total revenue was derived from the Vektis data. However, the Vektis data did not include the data of all insurers. The data were extrapolated to the whole population, based on the assumption that insurers for which no data were available did not have a deviant patient population, in terms of morbidity. For 2009 and 2010 the practice costs are based on the study of Significant[34]. For 2006 to 2008 we used the Significant study as basis and calculated the development in practice costs using the figures on GP practice costs published by Statistics Netherlands [35]. We did not extrapolate the figures based on national inflation figures, but based on the development as shown by the CBS data. These data are based on taxation data.

For all years in the study we included the additional remuneration for influenza vaccination for high-risk persons (those for whom influenza vaccination is recommended because of age or health condition), which contributes approximately 3% to the total revenues of the GP.

Table S6a. Remuneration in The Netherlands for 2000 - 2005 in Euro (for 2529 patients per GP)

| **Income components** | **2000** | **2001** | **2002** | **2003** | **2004** | **2005** |
| --- | --- | --- | --- | --- | --- | --- |
| Patients < 65 years, public health insurance | 90,381 | 99,580 | 103,919 | 108,474 | 108,474 | 109,613 |
| Patients > 65 years public health insurance | 15,936 | 17,558 | 18,336 | 19,144 | 19,144 | 19,314 |
| Private patients consultations | 31,058 | 34,626 | 38,542 | 42,505 | 42,505 | 42,505 |
| Private patients home visits | 5,317 | 5,929 | 6,599 | 4,105 | 4,105 | 4,105 |
| Private patients telephone visits + unknown + other | 6,802 | 7,583 | 8,441 | 17,089 | 17,089 | 17,089 |
| Influenza vaccination | 3,483 | 3,560 | 3,850 | 3,926 | 3,934 | 4,038 |
| **Total revenue** | 152,979 | 168,837 | 179,688 | 195,244 | 195,253 | 196,665 |
|  |  |  |  |  |  |  |
| **Practice costs** |  |  |  |  |  |  |
| Calculation using CPI from CBS and CBS-data from 2001 | 89,479 | 94,042 | 97,621 | 99,769 | 100,484 | 102,900 |
|  |  |  |  |  |  |  |
| **Total income** | **63,500** | **74,795** | **82,067** | **95,476** | **94,768** | **93,765** |

Peculiarities: The data for 2000-2005 may underestimate the income of GPs, since not all income components are included (such as driver’s licence examinations and other health tests). Furthermore, the assumption that there was no change in productivity may be incorrect in the light of population ageing, leading to a possible underestimation of the income in the years 2002-2005. Revenue from integrated care, which was introduced in 2010 formed 6% of the total revenue. This type of remuneration was excluded from the income calculations, because it is unclear which part of this figure is for the benefit of GPs and which part for other health care providers

Table S6b. Remuneration in The Netherlands for 2006-2010 in Euro

| **Income component** | **2006** | **2007** | **2008** | **2009** | **2010** |
| --- | --- | --- | --- | --- | --- |
| Capitation | 753,013,726 | 772,299,552 | 792,281,970 | 842,337,996 | 970,798,331 |
| Extra capitation | 51,881,722 | 62,439,712 | 79,632,413 | 107,062,319 | 117,328,009 |
| Consultations | 594,569,337 | 652,702,540 | 706,535,092 | 776,103,332 | 606,731,746 |
| Negotiated extra fees (*M&I*) | 114,885,218 | 149,481,071 | 194,355,672 | 245,355,083 | 231,448,246 |
| Total | **1,490,217,355** | **1,606,372,206** | **1,736,540,365** | **1,928,446,031** | **1,887,309,259** |
|  |  |  |  |  |  |
| Number of practice owner GPs[23] | 7,615 | 7,674 | 7,736 | 7,771 | 7,833 |
| Full-time %[23] | 0.81 | 0.81 | 0.81 | 0.83 | 0.83 |
| Number of FTE GPs (practice owners) | 6,168 | 6,216 | 6,266 | 6,450 | 6,501 |
| **Revenue per FTE GP from health insurance** | 245,511 | 263,343 | 282,917 | 305,563 | 296,291 |
|  |  |  |  |  |  |
| Influenza vaccination |  |  |  |  |  |
| Tariff | 9.18 | 9.33 | 9.60 | 9.88 | 10.00 |
| % Vaccinated persons | 18.1% | 18.6% | 21.8% | 22.4% | 21.3% |
| **Income from vaccination** | 3.888 | 4.038 | 4.878 | 5.163 | 4.979 |
| **Total revenue per GP** | **249,399** | **267,381** | **287,796** | **310,726** | **301,271** |
|  |  |  |  |  |  |
| **Practice costs** | **127,145** | **140,444** | **150,365** | **155,011** | **165,166** |
| **Total income per GP** | **122,254** | **126,936** | **137,430** | **155,715** | **136,105** |
|  |  |  |  |  |  |

*Sweden*

Data source: email contact with Malmö University Hospital, Faculty of Medicine (Campus Malmö) Lund University.

Calculations: Provided were the average yearly salaries of GPs.

Peculiarities: There are several different remuneration systems in place in Sweden, each county may have its own system.

Table S7. GP income in Sweden in Swedish Krona

| **year** | **income (SKr)** |
| --- | --- |
| 2000 | 520,164 |
| 2001 | 552,636 |
| 2002 | 581,832 |
| 2003 | 607,644 |
| 2004 | 626,868 |
| 2005 | 643,092 |
| 2006 | 665,424 |
| 2007 | 683,724 |
| 2008 | 719,424 |
| 2009 | 748,864 |

*United Kingdom (England)*

Data source: Data on GP-income came from publications of the Information Centre for Health and Social Care [39-42].

Calculations: until 2003, the Review Body on Doctors and Dentists Remuneration settled target income for individual GPs. In 2004 the payment system changed from being GP-based to being practice-based. GPs have the option to sign the GMS-contract (General Medical Service-contract) directly with the National Health Service, or sign a contract with the local health authorities (more specific: Primary Care Trusts), which is called the PMS-contract (Personal Medical Service contract). In this study, data on the remuneration of the GPs refer to GPs that have signed the GMS-contract and includes both dispensing and non-dispensing GPs.

Peculiarities: Dispensing GPs have a slightly higher income compared to non-dispensing GPs and GPs under the PMS-contract have a slightly higher income compared to GPs under the GMS-contract. The share of GPs under the PMS contract has increased from 25% in 2004 to about 50% in 2008. Thus there may be a slight underestimation of the GP income in the UK in our paper.

Table S8. GP income under the GMS contract in the United Kingdom (England) in Pounds

| **Year** | Total revenue | Practice costs | Income |  |
| --- | --- | --- | --- | --- |
| **2000** | 158,605 | 94,565 | 57,620 | * |
| **2001** | 166,965 | 100,851 | 64,040 | * |
| **2002** | 176,483 | 106,712 | 66,114 | * |
| **2003** | 190,942 | 113,345 | 69,771 | * |
| **2004** | 188,694 | 111,542 | 77,597 | * |
| **2005** | 217,097 | 120,775 | 96,322 | * |
| **2006** | 232,035 | 125,723 | 106,312 | * |
| **2007** | 231,525 | 131,201 | 103,530 | ** |
| **2008** |  |  | 100,324 | *** |
| **2009** |  |  | 99,200 | **** |

* GP Earnings and Expenses 2005/06 Final Report, Table S16a[40]

**GP-Earnings and expenses 2006/07 Final Report[41]

***GP-Earnings and expenses 2007/08 Final Report[42]

****GP earnings and Expenses 2008/09 Final report[43]

Table S9. Country score on the dimension of primary care strength [44]:

| **Total strength** |  |
| --- | --- |
| Strong: | Belgium, Denmark, Finland, Netherlands, UK |
| Medium: | Sweden, Germany, France |
|  |  |
| **Structure**: |  |
| Governance: |  |
| Strong: | Denmark, Netherlands, UK |
| Medium | Finland, Belgium, Germany, France, Sweden |
|  |  |
| Economic conditions: |  |
| Strong: | Germany, Belgium, Finland, Netherlands, UK |
| Medium: | France, Denmark, Sweden |
|  | |
| Workforce development: | |
| Strong: | Denmark, Finland, Netherlands, UK |
| Medium: | Belgium, France, Germany, Sweden |
|  |  |
| **Service delivery** |  |
| Access |  |
| Strong: | Denmark, Netherlands, UK |
| Medium: | Finland, Germany, Sweden |
| Weak: | France, Belgium |
|  |  |
| Coordination of care |  |
| Strong: | Denmark, Netherlands, Sweden, UK |
| Medium: | Belgium, Finland, France |
| Weak: | Germany |
|  |  |
| Continuity of care |  |
| Strong: | Denmark, Belgium, Germany |
| Medium: | Finland, France, UK |
| Weak: | Netherlands, Sweden |
|  |  |
| Comprehensiveness |  |
| Strong: | Belgium, Finland, France, Sweden, UK |

Table S10 GP income, revenue, practice costs (amount and share in revenue) in 2009 in pppUS$

|  | **Income** | **Revenue** | **Practice costs** | |
| --- | --- | --- | --- | --- |
|  |  |  | **pppUS$** | **Share** |
| Belgium | 103,733 | 158,510 | 54,777 | 35 |
| Denmark | 97,157 | 199,914 | 102,757 | 51 |
| Finland | 72,549 | - | - |  |
| France* | 69,169 | 127,406 | 58,236 | 46 |
| Germany | 145,346 | 300,923 | 155,577 | 52 |
| Netherlands | 185,187 | 369,536 | 184,349 | 50 |
| Sweden | 82,622 | - | - |  |
| UK* | 152,918 | 363,578 | 206,033 | 57 |

*France: 2005 and UK: 2007

Figure S1. Development in GP income compared to development in expenditure in basic medical care per capita in pppUS$


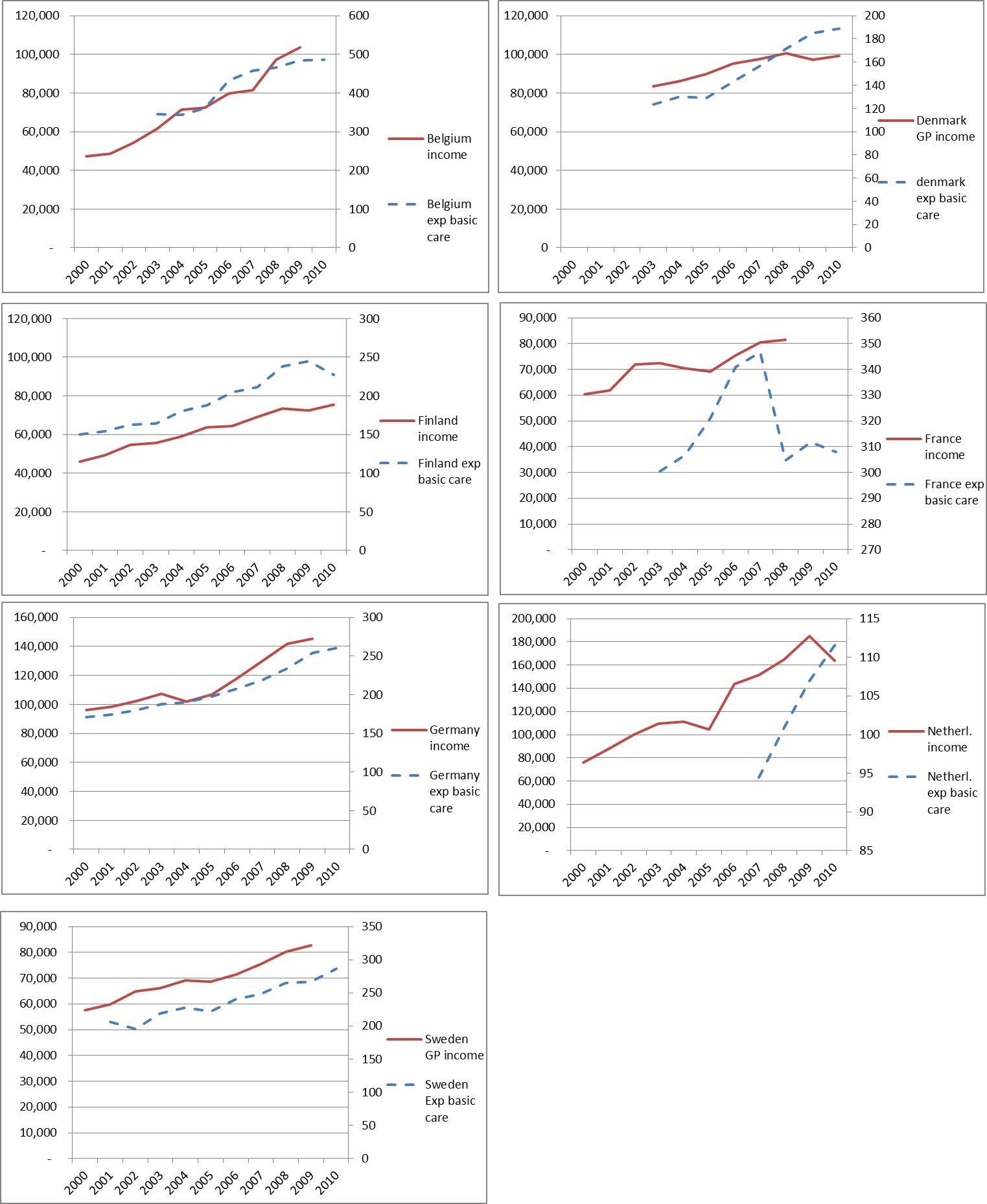


*France: a break in series for Expenditure on basic medical care is the cause of the sudden drop in expenditure.

*PPPUS$ conversion rates and Consumer Price Indices*

The pppUS$ conversion rates and the Consumer Price Indices were derived from the OECD health data files (the online version)

Table S11 PPPUS$ conversion rates for actual individual consumption

| Country | 2000 | 2001 | 2002 | 2003 | 2004 | 2005 | 2006 | 2007 | 2008 | 2009 | 2010 |
| --- | --- | --- | --- | --- | --- | --- | --- | --- | --- | --- | --- |
| Belgium | 0.8598 | 0.8645 | 0.8216 | 0.8596 | 0.8518 | 0.9001 | 0.8957 | 0.9029 | 0.8976 | 0.8989 | 0.8961 |
| Denmark | 8.2677 | 8.4745 | 8.0845 | 8.6046 | 8.5101 | 8.5946 | 8.6890 | 8.4782 | 8.3938 | 8.4909 | 8.4977 |
| Finland | 1.0087 | 1.0329 | 0.9928 | 1.0230 | 0.9950 | 0.9777 | 0.9916 | 0.9619 | 0.9482 | 0.9502 | 0.9461 |
| France | 0.8848 | 0.8652 | 0.8298 | 0.8831 | 0.8778 | 0.9238 | 0.8916 | 0.8815 | 0.8806 | 0.8720 | 0.8673 |
| Germany | 0.9241 | 0.9211 | 0.8890 | 0.8800 | 0.8624 | 0.8673 | 0.8314 | 0.8197 | 0.8078 | 0.8024 | 0.8011 |
| Netherlands | 0.8336 | 0.8511 | 0.8214 | 0.8722 | 0.8539 | 0.8966 | 0.8524 | 0.8392 | 0.8336 | 0.8409 | 0.8325 |
| Sweden | 9.0150 | 9.2236 | 8.9730 | 9.1819 | 9.0650 | 9.3833 | 9.3010 | 9.0772 | 8.9600 | 9.0637 | 9.1638 |
| UK | 0.6185 | 0.6119 | 0.5965 | 0.6189 | 0.6017 | 0.6365 | 0.6194 | 0.6368 | 0.6418 | 0.6487 | 0.6633 |
|  |  |  |  |  |  |  |  |  |  |  |  |
| Source: | OECD health data 2011, Online version access data 26-08-2011 | | | | | | | |  |  |  |

Table S12 Price Index Gross Domestic Product (2000=100)

|  | 2000 | 2001 | 2002 | 2003 | 2004 | 2005 | 2006 | 2007 | 2008 | 2009 | 2010 |  |
| --- | --- | --- | --- | --- | --- | --- | --- | --- | --- | --- | --- | --- |
| Belgium | 100 | 102.1 | 104.1 | 106.2 | 108.5 | 111.1 | 113.6 | 116.3 | 118.5 | 119.8 | 122.0 |  |
| Denmark | 100 | 102.5 | 104.9 | 106.6 | 109.1 | 112.2 | 114.6 | 117.2 | 121.7 | 122.2 | 126.1 |  |
| Finland | 100 | 103.0 | 104.3 | 103.6 | 104.1 | 104.6 | 105.5 | 108.7 | 110.6 | 111.7 | 114.1 |  |
| France | 100 | 102.0 | 104.4 | 106.4 | 108.1 | 110.3 | 112.9 | 115.7 | 118.7 | 119.3 | 119.9 |  |
| Germany | 100 | 101.2 | 102.6 | 103.9 | 104.8 | 105.5 | 105.9 | 107.9 | 109.0 | 110.5 | 111.2 |  |
| Netherlands | 100 | 105.1 | 109.1 | 111.5 | 112.3 | 115.0 | 117.1 | 119.2 | 122.0 | 121.8 | 123.8 |  |
| Sweden | 100 | 102.4 | 103.9 | 105.8 | 106.1 | 107.0 | 109.1 | 112.1 | 115.7 | 117.8 | 119.3 |  |
| UK | 100 | 102.1 | 105.3 | 108.5 | 111.3 | 113.5 | 117.0 | 120.5 | 124.1 | 125.9 | 129.5 |  |
|  |  |  |  |  |  |  |  |  |  |  |  |  |
| Source: | OECD health data 2011, Online version access data 26-08-2011 | | | | | | | | | | | |
